# Long-term efficacy and safety of spinal cord stimulation in patients with refractory angina pectoris

**DOI:** 10.1016/j.ijcha.2023.101194

**Published:** 2023-03-20

**Authors:** F.E. Vervaat, A. van der Gaag, K. Teeuwen, H. van Suijlekom, L. Dekker, I.F. Wijnbergen

**Affiliations:** a Department of Cardiology, Catharina Hospital, Eindhoven, the Netherlands; b Department of Anaesthesiology, Catharina Hospital, Eindhoven, the Netherlands

**Keywords:** Refractory angina pectoris, Spinal cord stimulation, Coronary artery disease

## Abstract

**Background:**

The number of patients with refractory angina pectoris (RAP), associated with poor quality of life, has been steadily increasing. Spinal cord stimulation (SCS) is a last resort treatment option leading to significant improvement in quality of life over a one year follow-up. The aim of this prospective, single-centre, observational cohort study is to determine the long-term efficacy and safety of SCS in patients with RAP.

**Methods:**

All patients with RAP who received a spinal cord stimulator from the period July 2010 up to November 2019 were included. In May 2022 all patients were screened for long-term follow-up. If the patient was alive the Seattle Angina (SAQ) and RAND-36 questionnaire were completed and if the patient had passed away cause of death was determined. The primary endpoint is the change in SAQ summary score at long-term follow-up compared to baseline.

**Results:**

From July 2010 up to November 2019 132 patients received a spinal cord stimulator due to RAP. The mean follow-up period was 65.2 ± 32.8 months. Seventy-one patients completed the SAQ at baseline and long-term follow-up. The SAQ SS showed an improvement of 24.32U (95% confidence interval [CI]: 18.71 – 29.93; p < 0.001).

**Conclusions:**

The main findings of the study show that long-term SCS in patients with RAP leads to significant improvement in quality of life, significant reduction in angina frequency, significantly less use of short-acting nitrates and a low risk of spinal cord stimulator related complications over a mean follow-up period of 65.2 ± 32.8 months.

## 1 Introduction

There is a growing number of patients with coronary artery disease (CAD) who have persisting angina pectoris despite optimal medical treatment known as refractory angina pectoris (RAP). It is estimated that 5 to 10% of patients with stable CAD have RAP [1]. In absolute numbers up to 1.78 million people in the United States have RAP with 50.000 to 100.000 new cases each year in the US and 30.000 to 50.000 new cases each year in Europe [2], [3]. The term refractory angina pectoris was first used by Mannheimer et al in 2002 and is defined as a chronic condition (more than three months) characterized by diffuse coronary artery disease in the presence of proven ischemia which is not amendable to a combination of medical therapy including the use of second- and third-line pharmacological agents, angioplasty including percutaneous coronary intervention (PCI) of chronic total occlusion or coronary bypass surgery [3], [4]. These patients have a poor quality of life due to the debilitating angina symptoms and are frequently admitted to hospitals, leading to a high use of health care resources.

The treatment options for patients with RAP are increasing but, as described in the recent European Society of Cardiology (ESC) guidelines for the management of chronic coronary syndromes, the level of evidence in support of the safety and efficacy varies from non-existent to promising [4]. One of these last-resort treatment options is spinal cord stimulation (SCS) with a Class of recommendation IIB and Level of evidence B [4]. The spinal cord stimulator is a device that consists of a lead in the higher thoracic epidural space (C7 – Th1) which provides stimulation at the level of the myocardial (afferent) neurons and is connected subcutaneously to an implantable pulse generator (IPG) in the abdomen or buttock. There are several proposed mechanisms of action: direct inhibition of pain, a decrease in sympathetic tone and changes in myocardial blood flow with a reduction in myocardial oxygen demand and an improvement in the coronary microcirculatory blood flow [5], [6].

Studies have repeatedly shown that SCS leads to a reduction in the number of angina episodes, less use of short-acting nitrates and a significant improvement in quality of life [7], [8], [9], [10], [11], [12]. The average duration of follow-up in these studies range from three months up to one year. Taking into consideration that patients with RAP have a one-year mortality rate of 3.9%, a nine-year mortality rate of 28.4% and an average age of 63.5 years at the time of diagnosis [3], a follow-up period of one year is relatively short. The question remains how patients with RAP who received SCS fare after their first year of treatment with regard to efficacy by looking at the number of angina episodes, use of short-acting nitrates and quality of life, as well as the long-term safety of SCS. The aim of this prospective, single-centre, observational cohort study is to determine the efficacy and safety of SCS in patients with RAP during a follow-up period of more than one year.

## 2 Methods

This is a prospective, single-centre, observational cohort study at the Catharina hospital in Eindhoven, the Netherlands. Approval for this study was given by the local institutional review board of the Catharina hospital. There was no patient and public involvement in the design and conduct of the study.

### 2.1 Patient selection

All patients with RAP who received a spinal cord stimulator at our hospital from the period July 2010 up to November 2019 were included if the following criteria were met: stable angina pectoris Canadian Cardiovascular Society (CCS) class III or IV (for at least three months), significant CAD with no revascularization options (coronary artery bypass grafting (CABG) and/or PCI) and optimal medical therapy. A complete overview of the in- and exclusion criteria is given in Table 1. All patients were assessed at baseline with regard to angina symptoms, quality of life and consumption of short-acting nitrates using the Seattle Angina Questionnaire (SAQ) and Research and Development Questionnaire-36 (RAND-36). The SAQ is a disease-specific, self-administered questionnaire used in patients with angina pectoris that has been applied in over 1800 studies [13]. The RAND-36 is a general health questionnaire that is one of the most widely applied health-related quality of life questionnaires in the world [14]. In addition, concomitant diseases, medication use at the time of spinal cord stimulator implantation and previous interventions (PCI and/or CABG) were systematically analysed.

**Table 1:** In- and exclusion criteria ACS = acute coronary syndrome, AP = angina pectoris, CAD = coronary artery disease, CAG = coronary angiogram, CCS = canadian cardiovascular society, FFR = fractional flow reserve, MCI = mild cognitive impairment, MIBI-SPECT = Methoxyisobutylisonitrile single photon emission computed tomography, MRI = magnetic resonance imaging, PET = positron emission tomography.

Inclusion criteria: - Refractory angina pectoris: ∘ Stable AP CCS class III or IV, during a minimum period of three months ∘ CAG (<12 months) showing significant CAD defined as at least one coronary artery stenosis of >75% or 50-75% with proven ischemia (see below), not suitable for revascularization as determined by the multidisciplinary heart team. ∘ Optimal anti-anginal medication; defined as at least use the maximal tolerable dose of: beta-blocker, calcium-channel blocker and/or short- and/or long-acting nitrate. If one group of anti-anginal medication is not used, the reason should be clear. - Proven ischemia: ∘ MIBI-SPECT: SDS of at leas 1 (1–4: mild ischemia, >4: moderate to severe ischemia) ∘ FFR: <0.80 with no interventional options as determined by the operator ∘ MRI perfusion: ≥1 segment of subendocardial hypoperfusion during stress perfusion, not present at rest and no matching fibrosis ∘ Myocardial perfusion PET: SDS of at least 1 - Age >18 years

Exclusion criteria: - ACS during three month period prior to screening - Life expectancy of <12 months - Inability to give informed consent - Spinal cord disease preventing correct positioning of the lead in the epidural space as determined by the operator - Anticoagulation therapy that cannot be stopped/bridged prior to spinal cord stimulator implantation - Inadequate paresthesia coverage, during implantation, of the thoracic region where the angina pectoris is localized - Pregnancy - MCI or dementia

In May 2022 all previously mentioned patients were screened for long-term follow-up. In those cases in which the patient was alive contact was sought by telephone and the patient was asked to complete both questionnaires (SAQ & RAND-36) and it was determined if the spinal cord stimulator was currently being used by the patient and if not what the underlying reason was. In those cases in which the patient had died the general practitioner or local hospital was contacted to determine the cause of death. In all cases data with regard to hospitalisations due to an acute coronary syndrome (ACS) and/or revascularisation were collected as well as data pertaining to the spinal cord stimulator, specifically infections and lead problems (dislocation and/or fracture and/or dysfunction). An ACS was defined as a high-sensitivity cardiac troponin (hs-cTn) T with at least one value above the 99th percentile of the upper reference limit and at least one of the following; (1) symptoms of myocardial ischemia, (2) new ischemic electrocardiographic (ECG) changes, (3) development of pathological Q waves, (4) imaging evidence of loss of viable myocardium or new regional wall motion abnormality or (5) intracoronary thrombus detected on angiography or autopsy, in accordance with the universal definition of myocardial infarction [15].

### 2.2 Endpoints

#### 2.2.1 Primary endpoint

The primary endpoint is the change in SAQ summary score (SS), defined as the average of the SAQ components physical limitations (PL), angina frequency (AF) and quality of life (QoL), at long-term follow-up compared to baseline.

#### 2.2.2 Secondary endpoints

The secondary endpoints are: (a) changes in the separate SAQ domains; PL, AF, QoL, treatment satisfaction (TS) and angina stability (AS) at long-term follow-up compared to baseline, (b) changes in use of short-acting nitrates at long-term follow-up compared to baseline, (c) changes in number of angina pectoris episodes at long-term follow-up compared to baseline, (d) changes in the summary scores and domains of the RAND-36 questionnaire, consisting of the physical health summary score, mental health summary score, physical functioning, role limitations due to physical health, role limitations due to emotional problems, vitality, mental health, bodily pain, social functioning and general health at long-term follow-up compared to baseline, (e) occurrence of an ACS and/or revascularisation at long-term follow-up and (f) device-related endpoints with regard to the spinal cord stimulator at long-term follow-up.

#### 2.2.3 Statistical analysis

To analyse the results the paired Student’s T-test was used. If values were not normally distributed the Wilcoxon signed rank test was used. The analysis was performed if both the baseline and long-term follow-up data from the same patient were available. To determine if the duration of follow-up had a significant effect on the results found the Pearson correlation coefficient was calculated for each dimension and summary score of the SAQ and RAND-36. A probability value of < 0.05 was considered statistically significant. The analysis was performed using SPSS version 28.0 for MacBook (SPSS, Inc., Chicago, Il, USA).

## 3 Results

### 3.1 Patient selection

**Fig. 1:**
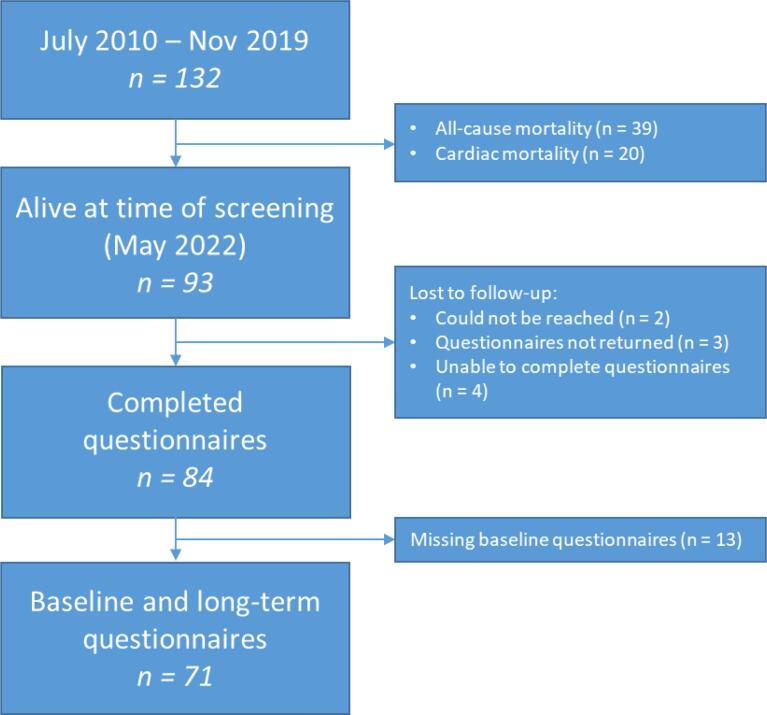
Flowchart of patient selection.

During the study period from July 2010 up to November 2019 a total of 132 patients received a spinal cord stimulator due to RAP. The mean follow-up period was 65.2 ± 32.8 months. At the time of long-term follow-up (May 2022) a total of 93 patients were alive (70.4%; of 132 patients). The all-cause mortality at long-term follow-up was 29.5% (39 of 132 patients) and the cardiac mortality 15.1% (20 of 132 patients) with an annual all-cause and cardiac mortality rate of 5.2% and 2.7% respectively. In total 84 patients returned the completed questionnaires. Of the nine patients who did not complete the questionnaires two patients could not be reached by telephone, three patients did not return the questionnaires and four patients had physical and/or mental health problems making it impossible to complete the questionnaires. For 13 patients the baseline questionnaires were not present and thus were not included in the final analysis of the questionnaires because both the baseline and long-term follow-up questionnaire had to be present (Fig. 1).

### 3.2 Patient characteristics

The patient characteristics are presented in Table 2. The average age at the time of spinal cord stimulator implantation was 67.3 ± 9.0 years and 112 (84.8%) of the patients were male. The majority of patients had undergone PCI and/or CABG previously (85.6% and 82.6% respectively) and 68.9% had three vessel disease. With regard to use of anti-anginal medication 82.6% was using beta-blockers, 66.7% calcium-channel blockers and 81.1% long-acting nitrates at the time of spinal cord stimulator implantation.

**Table 2:** Patient characteristics at baseline.

	n = 132
Age – yr	67.3 (±9.0)
Male – no. (%)	112 (84.8)
Smoking status – no. (%) Current tobacco user Previous tobacco user	20 (15.2) 67 (50.8)
Diabetes Mellitus – no. (%)	61 (46.2)
Dyslipidemia – no. (%)	83 (62.9)
Hypertension – no. (%)	73 (55.3)
Family history of coronary artery disease – no. (%)	86 (65.2)
Previous myocardial infarction – no. (%)	86 (65.2)
Previous PCI – no. (%)	113 (85.6)
Previous CABG – no. (%)	109 (82.6)
Aspirin – no. (%)	109 (82.6)
P_2_Y_12_ inhibitor – no. (%)	89 (67.4)
Oral anticoagulants – no. (%)	25 (18.9)
Beta-blocker – no. (%)	109 (82.6)
Calcium-channel blocker – no. (%)	88 (66.7)
Long-acting nitrates – no. (%)	107 (81.1)
Short-acting nitrates – no. (%)	117 (88.6)
Statins – no. (%)	121 (91.7)
Other lipid lowering agent – no. (%)	4 (3.0)
Left ventricular ejection fraction > 50% – no. (%)	110 (84.0)
Three vessel disease – no. (%)	91 (68.9)
Internal cardiac device* – no. (%)	24 (18.1)
* Pacemaker or internal cardiac defibrillator (ICD)	

## 4 Primary endpoint

### 4.1 Seattle angina questionnaire

A total of 71 patients completed the questionnaire at both baseline and long-term follow-up. For the SAQ SS there was a significant improvement of 24.32U (95% confidence interval [CI]: 18.71 – 29.93; p < 0.001). This was determined by less physical limitations (5.82U; 95% CI: 1.24 – 10.39; p = 0.013), reduced angina frequency (30.42U; 95% CI: 23.16 – 37.68; p < 0.001) and improved quality of life (26.77U; 95% CI: 20.17 – 33.37; p < 0.001). For the SAQ domain AS there was an improvement of 25.21U (95% CI: 16.47 – 33.96; p < 0.001) and for SAQ domain TS there was an improvement of 4.65U (95% CI: 0.08 – 9.22; p = 0.046) (Table 3). There was a moderate correlation between follow-up duration and the domains AS, AF and SS with *r* (67) = 0.248 (p = 0.040), *r* (69) = 0.285 (p = 0.016) and *r* (64) = 0.261 (p = 0.034) respectively. No significant correlation between follow-up duration and the other domains of the SAQ (Table 3).

**Table 3:** Outcomes Seattle Angina Questionnaire and RAND-36 questionnaire. Lower scores represent worse outcomes. Pearson’s correlation coefficient (*r*) between follow-up duration and the SAQ domains.

	n	Baseline	Follow-up	Change from BL	95%-CI	P value	Pearson’s correlation coefficient (*r*)	P value
*SAQ*								
Physical Limitation	71	35.0 ± 14.9	41.2 ± 20.3	6.16	1.63 – 10.68	0.008	0.093	0.443
Angina Stability	69	33.6 ± 26.2	58.8 ± 29.0	25.21	16.47 – 33.96	<0.001	0.248	0.040
Angina Frequency	71	38.1 ± 22.5	68.5 ± 26.0	30.42	23.16 – 37.68	<0.001	0.285	0.016
Treatment Satisfaction	67	72.0 ± 17.8	76.7 ± 17.3	4.65	0.08 – 9.22	0.046	0.144	0.244
Quality of Life	66	30.4 ± 18.4	57.1 ± 22.8	26.77	20.17 – 33.37	<0.001	0.147	0.237
Summary Score	66	32.3 ± 12.1	56.6 ± 21.0	24.32	18.71 – 29.93	<0.001	0.261	0.034

*RAND-36*								
Physical Functioning	64	33.3 ± 16.4	37.5 ± 24.0	4.21	−0.52 – 8.96	0.081	0.119	0.347
Role Physical	65	10.3 ± 26.1	27.3 ± 38.2	16.92	6.64 – 27.20	0.002	−0.050	0.692
Bodily Pain	64	38.4 ± 19.2	62.3 ± 21.5	23.88	17.48 – 30.28	<0.001	0.206	0.102
General Health	51	35.3 ± 16.1	35.6 ± 16.7	0.29	−4.73 – 5.32	0.907	0.054	0.704
Physical Health Summary Score	45	30.9 ± 13.8	41.7 ± 19.7	10.85	4.53 – 17.16	0.001	0.215	0.155
Social Functioning	48	42.1 ± 24.6	61.7 ± 29.1	19.53	10.86 – 28.19	<0.001	0.060	0.684
Role Emotional	64	48.9 ± 46.3	61.4 ± 44.1	12.50	−0.21 – 25.22	0.054	0.001	0.991
Mental Health	59	59.0 ± 13.6	66.5 ± 14.1	7.52	3.34 – 11.70	<0.001	0.106	0.425
Vitality	62	36.6 ± 19.6	47.4 ± 19.0	10.72	5.54 – 15.90	<0.001	0.218	0.089
Mental Health Summary Score	46	47.3 ± 20.5	60.6 ± 22.2	13.36	6.52 – 20.20	<0.001	0.032	0.832

## 5 Secondary endpoints

### 5.1 Short-acting nitrate use

**Fig. 2:**
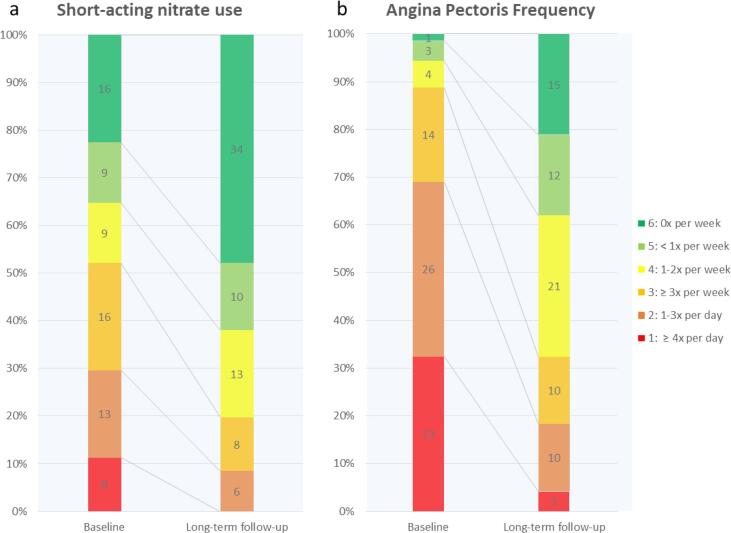
A. use of short-acting nitrates b. angina pectoris frequency – at baseline and at long-term follow-up. range 1 to 6; 1 = more than four times per day, 2 = one to three times per day, 3 = more than three times per week, 4 = one to two times per week, 5 = less than one time per week, 6 = no use of short-acting nitrate (a) / no angina pectoris episodes (b). Fig. 2: a. Use of short-acting nitrates b. Angina pectoris frequency – at baseline and at long-term follow-up.

There was a significant reduction in the frequency of short-acting nitrate use at long-term follow-up compared to the baseline from one to two times per week to less than one time per week (n = 71). Corresponding to a difference of 1.16U (range 1 to 6; 1 = more than four times per day, 2 = one to three times per day, 3 = more than three times per week, 4 = one to two times per week, 5 = less than one time per week, 6 = no use of short-acting nitrate) (95% CI: 0.75 – 1.58; p < 0.001) (Fig. 2a).

### 5.2 Frequency of angina pectoris episodes

There was a significant reduction in the frequency of angina pectoris episodes at long-term follow-up compared to the baseline from one to three times per day to one to two times per week (n = 71). Corresponding to a difference of 1.87U (range 1 to 6; 1 = more than four times per day, 2 = one to three times per day, 3 = more than three times per week, 4 = one to two times per week, 5 = less than one time per week, 6 = no angina pectoris episodes) (95% CU 1.43 – 2.31; <0.001) (Fig. 2b).

### 5.3 RAND-36 questionnaire

A total of 67 patients completed the questionnaire at both baseline and long-term follow-up. The physical health summary score improved by 10.85U (95% CI: 4.53 – 17.16; p 0.001) and the mental health summary score improved by 13.36U (95% CI: 6.52 – 20.20; p < 0.001). For the domain physical functioning there was an improvement of 4.21U (95% CI: −0.52 – 8.96; p = 0.081), social functioning improved with 19.53U (95% CI: 10.86 – 28.19; p < 0.001), role limitations due to physical health improved with 16.92U (95% CI: 6.64 – 27.20; p = 0.002), role limitations due to emotional problems improved with 12.50U (95% CI: −0.21 – 25.22; p = 0.054), vitality improved with 10.72U (95% CI: 5.54 – 15.90; p < 0.001), mental health improved with 7.52U (95% CI: 3.34 – 11.70; p < 0.001), bodily pain improved with 23.88U (95% CI: 17.48 – 30.28) and general health remained unchanged with 0.29U (95% CI: −4.73 – 5.32; p = 0.907) difference at long-term follow-up compared to baseline (Table 3). There was no significant correlation between follow-up duration and the domains of the RAND-36 (Table 3).

### 5.4 Cardiac events

In total 29 patients (22.0% of 132 patients) had an ACS during the follow-up period and 11 patients (8.3%) with an ACS underwent revascularisation. In addition 32 patients (24.2%) underwent revascularisation due to progressive angina pectoris symptoms during out-patient clinic follow-up. Leading to a total of 43 patients (32.5%) undergoing revascularisation during the follow-up period.

### 5.5 Device-related events

During the follow-up period 13 patients (8.3% of 132 patients) had the spinal cord stimulator switched off for a variety of reasons; five patients experienced progression of symptoms despite SCS, six patients had a reduction of symptoms due to additional treatment (1 transcatheter aortic valve replacement (TAVR), 1 PCI, 2 CABG, 1 shockwave therapy & 1 adjustment of medication), one patient had a lead dysfunction and in one patient the IPG was empty, due to dementia no new IPG was implanted. Three patients (2.3%) had the spinal cord stimulator removed; one patient due to incompatibility of the spinal cord stimulator with an MRI, one patient due to pain at the site of the IPG (left buttock) and one patient due to recurrent IPG pocket infections. Four patients (3.0%) underwent revision of the IPG pocket and/or lead. Two patients (1.5%) had a spinal cord stimulator related infection; in one patient this led to the removal of the spinal cord stimulator and in the second patient it was a superficial infection of the IPG pocket which was successfully treated with antibiotics.

## 6 Discussion

The main findings of the study show that long-term SCS in patients with RAP leads to significant improvement in quality of life, a significant reduction in angina frequency, as well as significantly less use of short-acting nitrates over an mean follow-up period of 5,4 years.

To our knowledge this study is the first to publish results regarding the efficacy and safety of SCS in patients with RAP with a follow-up period of more than one year [10], [12], [16]. Patients in our patient population are relatively young at the time of spinal cord stimulator implantation, 67.3 (±9.0) years. Based on current data it is known that patients with RAP have a good long-term life expectancy with an annual mortality rate of <4% a year [3] emphasizing the relevance of more long-term data.

To determine the long-term effect of SCS the SAQ was applied. The SAQ was first introduced in 1994 and the SAQ summary score (SS) was created in 2014 [17], [18]. It is a disease specific questionnaire that can be used in patients with CAD and is designed to quantify the quality of life, symptoms and functioning in these patients. The SAQ consists of five domains; physical limitations (PL), angina frequency (AF), angina stability (AS), treatment satisfaction (TS) and quality of life (QoL), with scores ranging from 0 to 100. Higher scores equal better performance in each of these domains. The primary endpoint of the study was the change found in the SAQ SS which was an increase of 24.32U at long-term follow-up compared to baseline. The summary score was developed by Chan et al and is the average of changes occurring in the domains physical limitations, angina frequency and quality of life [18]. More than five points change in the SAQ SS is considered to be clinically relevant which is the case in our study [19], [20]. A difference of eight points is considered to be the minimum clinically important difference for the domains AF, PL and QoL and for the domain TS this is five points [17], [21]. In our study a clinically important difference was shown in the domains AF (30.42U difference), QoL (26.77U difference) and the SS, but not in the domains PL (5.82U difference) and TS (4.65U difference). A possible explanation for the relatively small difference in the domain PL could be due to the fact that other health issues besides angina pectoris could lead to impairment of specific activities. With regard to the TS domain it can postulated that patients were already satisfied with their current treatment and this has remained so over the follow-up period. For the domains AS, AF and SS there was a moderate correlation with the follow-up duration showing that a longer follow-up period leads to even more improvement in these domains. Summarizing the SAQ results from this study show that patients with RAP using SCS have a significant long-term improvement in angina pectoris symptoms and quality of life.

The RAND-36 questionnaire containing eight domains is a general health questionnaire frequently used in clinical trials [14]. With regard to clinically significant changes in the domain scores more than five points change, but also at least ten points change have been described as being clinically significant [20], [22]. In our study the two summary scores, physical health and mental health, both increased by more than ten points, 10.85U and 13.36U respectively. Although this was not true for all of the separate domains with physical functioning (3.93U difference), general health (-0.09U difference) and mental health (7.14U difference) scoring less than ten points difference at long-term follow-up. This is likely due to the fact that it is a questionnaire used to look at the patients’ general health and can be influenced by other medical conditions that have a negative effect on the patients’ perception of his general health. This leads to a lower score despite the fact that the angina pectoris symptoms could have improved over the same period. In conclusion the RAND-36 results from our study show a significant general improvement in the health-related quality of life during long-term follow-up in patients with RAP using SCS.

The clinical endpoints show that 22.0% (n = 29) of patients with RAP and SCS had an ACS and 32.5% (n = 43) underwent revascularisation (24.2% due to progressive angina pectoris and 8.3% due to an ACS) during the follow-up period. Other studies with a shorter follow-up period, ranging from six up to twelve months, reported occurrence of an ACS in 7.4% up to 10.3%, and revascularisation ranged from 3.3% up to 11.5% during follow-up [9], [10], [12], [23]. These numbers show that the occurrence of a cardiovascular event is to be expected. CAD is a chronic disease which is often progressive and leads to future cardiovascular events such as an ACS. The variety of treatment options are aimed at disease stabilization, but cardiovascular events can still occur in patients with RAP despite optimal treatment as is seen in this study [4].

Based on the device-related events, 3.0% revision of lead and/or IPG pocket and 1.5% spinal cord stimulator related infection, it can be concluded that SCS is a safe treatment modality with a low rate of events during the follow-up period. In total three patients had the spinal cord stimulator removed with one patient due to recurring infections. The device-related event rates are comparable to those described in a review aimed specifically at device-related events in patients with RAP and SCS. The reported rate was 1% for device-related infection and 7.8% for lead migration or fracture [24]. Reported rates of device-related infections in individual studies ranged from 0% up to 13.3% and led to explantation of the spinal cord stimulator in some cases [6], [9], [12], [23], [25], [26], [27]. With regard to lead dislocation/migration/fracture the reported rates in the individual studies ranged from 1.9% up to 8.8% [6], [9], [12], [23], [25], [26], [27]. An important factor in the occurrence of device-related events is operator experience. It has been proven that the experience of the operator significantly impacts the rate of device-related events [28]. Therefore it is important that spinal cord stimulator implantations take place at centres with ample experience to ensure a low rate of device-related events. The consequence is that spinal cord stimulator implantation cannot occur at all centres, but should be centred in selected, specialized centres.

In total 13 patients (8.3%) had stopped using SCS at long-term follow-up. For the majority of these patients (6 out of 13) the reason to discontinue SCS was significant reduction of angina pectoris after CABG, PCI, shockwave therapy, TAVR or medication adjustment. Other studies have reported 2.5% up to 7.7% of patients discontinuing SCS or even explanting the spinal cord stimulator [10], [23]. An important side note is that the reason for explanting the spinal cord stimulator is most likely due to a device-related infection. In addition the majority of studies had a follow-up period of up to one year, decreasing the likelihood of patients discontinuing SCS.

An important limitation of the study is the variation in follow-up duration for each patient due to the fact that the long-term data was gathered at one point in time, but the date of implantation varied per patient. To compensate for this limitation the Pearson’s correlation coefficient was applied to correct for this variation. Only a moderate correlation was found for three domains in the SAQ (AS, AF and SS) showing only a small effect of the variation in follow-up duration.

Due to the observational character of the study the results reported with regard to the questionnaire related endpoints, including the primary endpoint (SAQ summary score) should be cautiously interpreted. Only those patients with a relatively favourable course of the disease who were still alive at the time of long-term follow-up were able to complete the questionnaires. It remains unknown what the changes in these endpoints were for the whole cohort of patients included at baseline.

An inherent limitation of the study is the absence of a control group due to the fact that it was an observational cohort study. As has been previously reported randomised controlled trials (RCT) in this patient population is difficult due to slow inclusion rates leading to small samples varying from 12 to 32 patients [7], [8]. Another important factor in the relatively few RCTs performed on this subject is the difficulty of truly blinding patients. This is due to the fact that paraesthesia are felt by the patient when the spinal cord stimulator is switched on making blinding impossible. In recent years newer forms of stimulation have become available, such as high density (HD) or burst stimulation, which are paraesthesia free modes of spinal cord stimulation. In other pain syndromes these new forms of spinal cord stimulation have proven to be equally effective and even superior to conventional spinal cord stimulation [29]. The availability of these new paraesthesia free modes of spinal cord stimulation will make proper blinding in future studies possible. With regard to future studies the importance of additional research in patient with RAP was confirmed in the recent ESC guidelines management of chronic coronary syndromes. In the guidelines it was concluded that additional research is necessary to determine the exact role of the various treatment modalities, such as SCS, for patients with RAP. To provide more clarity on this point a double-blind, cross-over, placebo-controlled, single-centre RCT, the SCRAP trial, is being undertaken (NCT04915157). The aim of this study is to determine if SCS leads to a significant reduction in myocardial ischaemia in patients with RAP using the PET perfusion scan whilst applying high density spinal cord stimulation to ensure proper blinding.

## 7 Conclusion

This prospective, single-centre, observational cohort study has shown that SCS in patients with RAP is effective and safe over a long follow-up period of 68.7 ± 33.7 months with a significant and sustained improvement in quality of life, less episodes of angina pectoris and less use of short-acting nitrates.

## Declaration of Competing Interest

The authors declare that they have no known competing financial interests or personal relationships that could have appeared to influence the work reported in this paper.
